# Treatments of Interest in Male Breast Cancer: An Umbrella Review

**DOI:** 10.3390/jpm15020066

**Published:** 2025-02-11

**Authors:** Stefano Spinaci, Luca Arecco, Agnese Anedda, Lucia Martino, Emma Firpo, Matteo Ghilli, Matteo Lambertini, Giulia Ferrarazzo

**Affiliations:** 1 Breast Unit, Department of Surgery, ASL3, Ospedale Villa Scassi, 16149 Genova, Italy; stefano.spinaci@asl3.liguria.it; 2 Department of Internal Medicine and Medical Specialties (DIMI), School of Medicine, University of Genova, 16132 Genova, Italy; arecco.luca@hubruxelles.be (L.A.); matteo.lambertini@unige.it (M.L.); 3 Academic Trials Promoting Team, Institut Jules Bordet, Hôpital Universitaire de Bruxelles (HUB), Université Libre de Bruxelles (U.L.B.), 1050 Brussels, Belgium; 4 Department of Radiology, ASL3, Ospedale Villa Scassi, 16149 Genova, Italy; agnese.anedda@asl3.liguria.it (A.A.); lucia.martino@asl3.liguria.it (L.M.); 5 Breast Surgery, Department of Surgery, ASL3, Ospedale Villa Scassi, 16149 Genova, Italy; emma.firpo@asl3.liguria.it; 6 Breast Centre, Breast Surgery, University Hospital, 56126 Pisa, Italy; m.ghilli@ao-pisa.toscana.it; 7 Department of Medical Oncology, U.O. Clinica di Oncologia Medica, IRCCS Ospedale Policlinico San Martino, 16132 Genova, Italy; 8 Nuclear Medicine, ASL3, Ospedale Villa Scassi, 16149 Genova, Italy

**Keywords:** male breast cancer, treatment, systematic review

## Abstract

**Background:** Male breast cancer (MaBC) is a rare disease and due to its rarity and the lack of specific protocols for its management, treatment algorithms are extrapolated from female breast cancer (FBC). To optimize MaBC treatment, we conceived an umbrella review with the aim of supplying an evidence-based summary of systematic reviews published about this topic in the last twenty years. **Methods:** This umbrella review was performed according to a predefined protocol (PROSPERO number CRD42024574299). We performed a literature search of the PubMed and Cochrane Libraries databases and we considered systematic reviews on MaBC treatment published from 2004 to 2024. We evaluated relevant treatments in the management of MaBC, including surgery, radiotherapy, and systemic treatments. We conducted the quality assessment according to A MeaSurement Tool to Assess systematic Reviews version 2 (AMSTAR-2), and the description of the main findings of eligible articles. **Results:** Seven systematic reviews were selected and the main findings were compiled. Breast-conserving surgery is a reasonable treatment approach and, in selected cases, equivalent in terms of safety and survival outcomes compared to mastectomy. Sentinel lymph node biopsy represents a successful surgical practice with similar accuracy compared to female cases. Adjuvant radiotherapy improves overall survival in MaBC patients following partial mastectomy and after radical mastectomy, in case of involved nodes. Finally, Tamoxifen is associated with an improvement of survival outcomes; aromatase inhibitor and gonadotrophin-releasing hormone should be used only in case of contraindications to tamoxifen. **Conclusions:** Further research and improved guidelines for MaBC treatment should consider these evidence-based data.

## 1. Introduction

Male breast cancer (MaBC) is a rare disease and accounts <1% of all breast cancers [1,2] but its incidence is rising [3]. Several epidemiological and genetic risk factors are associated with MaBC: family history, black race, obesity, breast or chest irradiation, use of estrogen or testosterone, orchitis/epididymitis, finasteride, Klinefelter’s syndrome, and pathogenic variants in susceptibility genes (e.g., BRCA1/2, CHEK2 and PALB2) [4,5,6,7,8,9].

MaBCs are invasive breast cancers in 90% of the cases, with an 80% of ductal histology, 5% papillary, and only 1% lobular histology, while medullary, mucinous, and tubular represent other less frequent subtypes. The non-invasive MaBCs comprise 10%, with a majority of ductal carcinoma in situ (DCIS) [10,11]. Most MaBCs are estrogen and progesterone receptor-positive while there is a greater variability for human epidermal growth factor receptor 2 (HER2) overexpression (2–42%) [12].

Because of the rarity of this disease and the lack of a specific protocol for its management, diagnostic standards and therapeutic schemes of early-stage and metastatic MaBC are extrapolated from female breast cancer (FBC) [9,13,14,15,16]. However, MaBC differs in clinical, pathological, and biological features and it is usually diagnosed later than in female patients, resulting in advanced stages at diagnosis (i.e., larger sizes and extensive nodal involvement) [17] and with a worse prognosis [18]. Due to these reasons, this kind of cancer is very challenging. These differences must be considered: awareness, education, and the development of specific guidelines for this male cancer are essential to ensure early detection as well as diagnosis and appropriate treatment.

Total mastectomy represents the most frequent surgical option for MaBC while breast-conserving surgery (BCS) is reserved just for selected cases [18,19]. A challenge is posed by sentinel lymph node biopsy (SLNB) due to gender-based anatomical differences [20,21] but its feasibility has been demonstrated by several small studies [22,23,24,25,26,27,28,29].

Post-mastectomy radiotherapy is usually recommended at all MaBC stages due to the smaller breast size and the frequent limited extent of resection margins [30,31,32,33,34,35,36,37].

Frequently, MaBC patients are estrogen receptor (ER)-positive and adjuvant endocrine therapy (ET) is mandatory [1,38,39,40,41,42,43]. The role of neoadjuvant and adjuvant chemotherapy, and the therapeutic management of metastatic MaBC in general, must be further evaluated [42,44]. However, evidence regarding MaBC treatments is limited; therefore, we performed this umbrella review with the aim of summarizing different aspects of MaBC treatment, including surgery approaches, radiotherapy, and systemic treatments, suppling an evidence-based summary of systematic reviews published about this topic in the last twenty years.

## 2. Materials and Methods

### 2.1. Search Strategy and Selection Criteria

The present umbrella review (or systematic review of systematic reviews) was performed according to the Preferred Reporting Items for Systematic Reviews and Meta-Analyses (PRISMA) guidelines [45,46].

We created a search string combining keywords and Boolean operators: (A) “male” OR “men” OR “man” AND (B) “breast” AND (C) “cancer” AND (D) “systematic review” and we performed a literature search of the PubMed and Cochrane Library databases with no language restrictions.

To be included in this umbrella review, studies should be systematic reviews, with or without meta-analyses, published from 2004 to September 2024 (the last update of the search was 31 October 2024) and report the oncological outcomes of MaBC treatments (i.e., surgery, radiotherapy, and systemic therapies).

Relevant articles were cross-referenced to confirm that all possible pertinent records were identified.

The quality assessment of the data was performed according to a MeaSurement Tool to Assess systematic Reviews, version 2 (AMSTAR-2).

This work is registered with the International Prospective Register of Systematic Reviews (PROSPERO) registration number CRD42024574299, and the full protocol is available on the PROSPERO website.

### 2.2. Study Objectives

This umbrella review aims to evaluate the systematic reviews of retrospective or prospective case–control or cohort studies and clinical trials reporting and/or comparing the different treatment outcomes of different breast surgery techniques (mastectomy or BCS), sentinel lymph node biopsy (SLNB), radiotherapy and adjuvant systematic treatment (chemotherapy and ET) in men treated for early and metastatic BC, in terms of oncological outcomes (i.e., disease-free survival, recurrence rate, objective response rates, BC-specific survival, overall survival).

### 2.3. Data Analysis

The systematic literature search was carried out independently by two authors (GF and SS), and any discrepancies were solved by discussion with a third author (LA).

For every selected systematic review, information about the authors, year of publication, number of original articles, included MaBC patients, and main findings was collected.

## 3. Results

Articles retrieved

**Figure 1:**
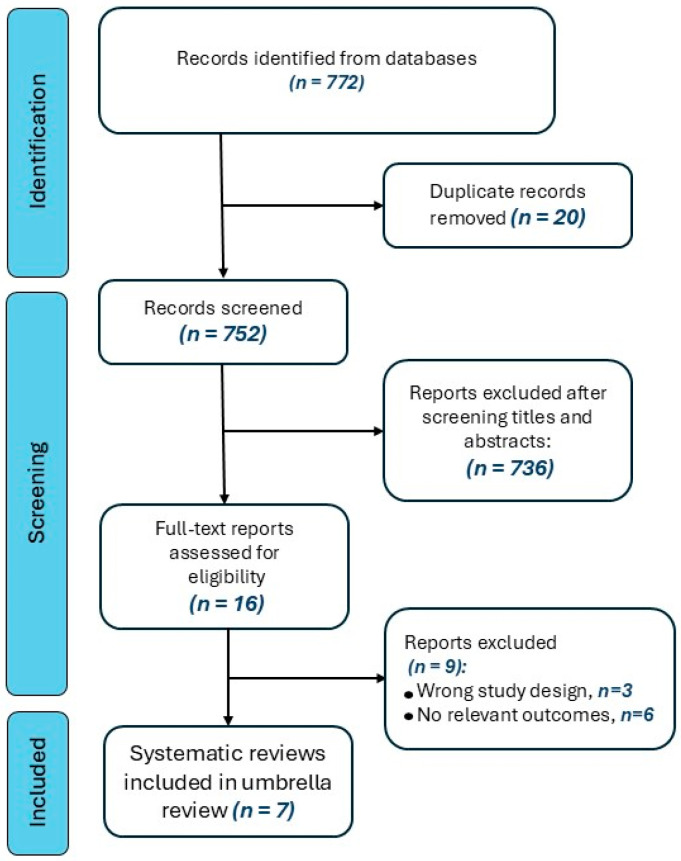
Flow of records searched according to the present systematic review.

According to the search strategy, 772 records were retrieved. Of these records, seven systematic reviews, according to the predefined inclusion and exclusion criteria [47,48,49,50,51,52,53], were included in this umbrella review (Figure 1).

The systematic reviews included a minimum of 8 and a maximum of 59 studies, while the number of patients included ranged from as few as 164 to 80,219.

Main characteristics and findings of the selected articles are presented in Table 1.

The quality assessment of the included systematic reviews is available in the Supplementary Materials (Table S1).

### 3.1. Surgery

#### 3.1.1. Breast-Conserving Surgery vs. Mastectomy

Five systematic reviews reported mastectomy and BCS survival outcomes and, most of them, a comparison regarding these data [47,48,49,51]. The outcomes of interest were disease-free survival (DFS), disease-specific survival (DSS), local recurrence (LR), and overall survival (OS).

The first systematic review [47] involving a cohort 10,965 patients, showed that radical mastectomy has been replaced by the modified radical mastectomy, over the last decades, and no differences in locoregional relapse rates have been demonstrated. Partial mastectomy has been often performed on patients with limited disease for esthetic reasons and greater tolerability but also, as palliative, in older men with advanced BC.

Later, De La Cruz et al. [51] evaluated eight prospective studies conducted on 859 males treated with BCS among a total cohort of 5864 patients. Stage II BC has proven to be the most frequent clinical stage in four of the five studies where the clinical stage has been reported. Among 116 patients treated with BCS, low rates of LR weighted average (9.9%) were reported. Among 14 cases of BCS, the DFS weighted average was 85.6%. Finally, among 743 patients treated with BCS, the 5-year OS weighted average was 84.4%.

In the systematic review by Sauder et al. [48], nine studies have been included (of which three studies were also included in the analysis of De La Cruz et al.) reporting at least one survival outcome among DFS, DSS, and OS. They found an equivalent 5-year DSS and OS for mastectomy and BCS, after adjusting for some patients’ features (age, year of diagnosis, stage, histology, and radiation therapy). Local recurrence or DFS were reported for a total of 986 patients. No difference was reported in local recurrence rates for BCS and mastectomy among 566 men. On a cohort of 420 patients with stage I disease, a higher 5-year DFS was found for patients treated with BCS and radiation therapy, compared to patients who underwent mastectomy alone (78% vs. 45%).

Finally, Lin et al. [49] included another seven studies on BCS vs. mastectomy: 2973 patients underwent BCS out of a total cohort of 14,061 patients, with no significant differences in 5- and 10-year OS rates between the groups. In two studies, in patients treated with BCS, equivalent DFS and local control and a lower incidence of post-surgery complications were recorded.

Overall, the authors concluded that BCS and mastectomy have equivalent survival outcomes and emphasized the oncologic safety of the first type of surgical approach.

Rutherford et al. [50] evaluated 39,529 patients assessed from 59 studies published from 1992 to 2021. These trials reported that mastectomy has been the most frequent technique used (89.6%); in these patients, 5- and 10-year DFS was 66.8% and 54.5%, respectively, while 5- and 10-year OS was 72.7% and 50.7%, respectively.

#### 3.1.2. SLNB 

Lin et al. [49], included eleven studies evaluating identification rates of SLNB in 213 cases of MaBC. Dual mapping techniques have been used in most studies. This kind of surgery proved to be successful and accurate in the majority of cases, showing, at the meta-analysis, similar accuracies to female cases (an identification rate of 97.4% and a false-negative rate of 7.4%).

A systematic review and meta-analysis by Parpex et al. [52] was completely focused on evaluating the accuracy of the SLNB in MaBC cases. Twelve retrospective studies (some of which were already included in the study by Lin et al. [49]) on men with negative preoperative axillary evaluation and primary surgery were included. On a total cohort of 164 patients, the pooled estimation of the SLNB identification rate was 99.0%. The false negative rate of this practice, evaluated on 50 patients, was 0%. These findings suggested that, in the selected clinical conditions described above, SLNB was feasible and efficient. This research also supported the extemporaneous histologic evaluation of SLNB in MaBC to quickly perform ALND in case of a confirmation of axillary lymph node metastases.

### 3.2. Radiotherapy

Three systematic reviews aimed to evaluate the impact of radiotherapy on local control and survival outcomes in MaBC patients.

In their systematic review [47], Jardel et al. evaluated any possible benefits of adjuvant locoregional irradiation systematically proposed. They found 14 studies comparing survival outcomes with and without post-mastectomy radiotherapy.

The rate of men that underwent radiotherapy varied between 3% and 100% and most studies did not clarify the selected target volumes. A statistical benefit in OS rates was found in stage I and stage III patients who received post-mastectomy radiotherapy, compared to men not treated with radiotherapy after surgery (i.e., stage III 10-year OS: 26.4% in patients treated with radiotherapy vs. 11.9% in patients without adjuvant radiotherapy) was reported. A trend in favor or adjuvant radiotherapy has been shown in case of close or unknown margins.

Of a cohort of 1933 patients, no difference in 5-year OS was found between men receiving adjuvant radiotherapy and men who did not receive it (78% vs. 77%, respectively, *p* = 0.371), but in 315 cases of the same cohort, matched by age, race, T- and N-stage, histological grade, and ER status, adjuvant radiotherapy gave advantages in 5-year OS (83% vs. 54%, *p* < 0.001). In case of positive lymph nodes, the advantages of radiotherapy became significant; compared to cases with no adjuvant radiotherapy: 5-year OS 79% vs. 72%, *p* = 0.05 with 1–3 positive nodes, and 73% vs. 53%, *p* < 0.001 with ≥4 nodes). Studies on 387 patients, regarding the comparison of OS between MaBC and FBC, were additionally presented [47].

Later, Lin et al. [49] also analyzed fifteen studies (six of them have already been included in the systematic review by Jardel et al. [47]), evaluating the effects of post-mastectomy radiation therapy. Among 11,392 men, 3648 were treated with chest wall and/or node post-mastectomy radiotherapy, while 7744 MaBC patients were treated with surgery alone. The disease stage was more advanced in the post-mastectomy radiation group. OS significantly increased in cases with radiation therapy. The 5-year OS, DFS, and LRFS also showed an increasing, although non-significant, trend in the post-mastectomy radiation group [49].

In their very recent review, in which data from 80,219 MaBC patients were quantitatively evaluated, Colciago et al. [53] observed a statistically notable risk of death reduction when the therapeutic algorithm of MaBC patients included RT, with a pooled aHR = 0.73 (95%CI: 0.66 to 0.81) for OS.

Sauder et al. [48] showed, among the majority of patients who underwent BCS, low rates of radiation therapy compliance (27–46%). Post-mastectomy radiotherapy rates were between 8% and 61%.

### 3.3. Systemic Treatments

Adjuvant chemotherapy and ET have also been more often administered over the past decades in male patients.

Jardel et al. [47] found that, in a cohort of 10,965 patients, the rate of men treated with chemotherapy and ET varied between 3% and 85% (mean = 26%) and between 7% and 92% (mean = 45%), respectively. Between 1990 and 2005, 72% of 489 MaBC patients received ET (85% tamoxifen and 12% aromatase inhibitors) and this percentage increased over time (from 57% in 1988–1995 to 82% in 1988–1995, *p* < 0.0001); moreover, 34% of patients received adjuvant chemotherapy (anthracyclines), also with an increase in this case from 25% in 1988–1995 to 37% in 1996–2005 (*p* = 0.029).

Rutherford et al. [50], in a cohort of 39,529 MaBC patients mostly (84%) affected by ER-positive disease, reported that ET was heterogeneously administered to an average of 58% treated men.

Lin et al. [49] analyzed ten cohort studies conducted on a total of 11,229 patients, comparing the efficacy of various ETs. Most patients were hormone receptor-positive (HR+), while not many patients had HER2-positive disease. Seven studies evaluated the beneficial effects of tamoxifen: in the tamoxifen group, the performed meta-analysis revealed a significantly increased DFS (HR 0.44, 95% C.I. 0.28 to 0.69) and OS (HR 0.62, 95% C.I. 0.41 to 0.95) among patients treated with tamoxifen compared to patients who were not. Tamoxifen treatment was also favored by 5-year OS (OR 1.76, 1.60 to 1.94), 10-years OS (OR 1.87, 95% C.I. 0.98 to 3.54), 5-year DFS (OR 2.72, 95% C.I. 1.57 to 4.70), and 10-year DFS (OR 3.34, 95% C.I. 1.95 to 5.71). Two studies compared the efficacy of tamoxifen with aromatase inhibitor, revealing a significant increase in 5-year OS for tamoxifen against aromatase inhibitor alone (OR 2.35, 95% C.I. 1.17 to 4.74), while one study compared the survival outcomes of the aromatase inhibitor with gonadotrophin-releasing hormone (GnRH) versus the aromatase inhibitor alone, in metastatic MaBC; the resulting data favored the use of the combined treatment (OS: OR 2.40, 95% CI 0.83 to 6.97).

## 4. Discussion

MaBC therapy is still challenging and the treatment procedures for this rare cancer, but with a rising incidence, are still debated. The present umbrella review aims to summarize the major evidence-based information about the different possible steps of MaBC treatment, taken from systematic reviews published in the last twenty years: breast and axillary surgery, the role of the radiation therapy, and the benefits of adjuvant systematic treatments.

The data obtained from the systematic reviews conducted in this field prove that:

(1) BCS enables high survival rates and low recurrence risks. BCS survival outcomes, such as OS, DFS, and LR, are equivalent to those of mastectomy, if not better, especially if the tumor is <2 cm, and in other selected cases. Moreover, radiotherapy, which reduces the recurrence risk as already discussed, conventionally follows BCS. These findings suggest that breast conservation is a reasonable alternative treatment approach in MaBC cases without clear nipple or areolar involvement. Finally, the preservation of healthy breast tissue would allow better cosmetic outcomes, improving self-image in men;
(2) SLNB is characterized by low false-negative rates, without any infection at the injection site (subareolar or peritumoral). Furthermore, similar accuracy rates to those of FBC have been proven. Findings in the literature support the feasibility of SLNB and encourage this practice in all MaBC with clinically negative axilla but further research is needed. Indeed, several large trials compared the survival rates of SLNB and ALND in FBC. For example, the ACOSOG Z0011 and EORTC AMAROS trials [54,55] conducted on cT1-2 FBC with postoperative radiotherapy to the breast and/or the region, with one or two positive SLNs, caused a decline in ALDN use. The available data proved the diagnostic accuracy of SLNB in men, but the relatively small sample sizes and the retrospective design of the included studies highlight the need for larger prospective trials to fully evaluate the long-term oncologic outcomes of SLNB and to establish its role in the management of MaBC. Recently, larger retrospective studies [28,56] have been published, but as highlighted by some authors [27,57], there is a need to further study the clinical impact of SLNB in different subpopulations of men. It is crucial to effectively translate SLNB benefits into MaBC treatment by avoiding ALDN, in those cases in which SLNB has proved its high diagnostic accuracy and comparable survival rates. The challenges are represented by the different tumor presentations, pathological and biological features, and the later diagnosis compared to female patients, which may influence the effectiveness and reliability of SLNB in MaBC;
(3) Adjuvant radiotherapy improves MaBC local disease control and OS. It is mandatory after partial mastectomy. Following radical mastectomy, chest wall radiotherapy and nodal radiotherapy have to be considered in case of involved nodes; radiotherapy can improve the local control of the disease and OS in MaBC even in this case. However, evidence is not sufficient regarding pN0 MaBC patients. Many MaBC patients choose to undergo chest wall radiotherapy despite total mastectomy, unlike female patients without advanced disease; this treatment practice could be usually opted for because of MaBC’s proximity to surgical margins and/or muscle tissue. Evidence-based findings prove that MaBC and FBC are different and more specific guidelines for men should be developed. Furthermore, an accurate definition of target volumes is often not possible. In order to avoid unnecessary toxic effects, the identification of cases in which radiation treatment is really useful is fundamental;
(4) Adjuvant endocrine therapy remains mandatory because most MaBCs are HR+. Tamoxifen, in accordance with current international guidelines [1,2], has been proved to be associated with an improvement in survival outcomes, showing it to be superior with respect to an aromatase inhibitor, mainly in cases where therapy is extended for at least 5 years. Aromatase inhibitors and gonadotrophin-releasing hormones (GnRHs) should be used in case of contraindication to tamoxifen. Further research about the side effects of Tamoxifen (e.g., increased weight, flushing, sexual dysfunction, venous thromboembolism), which often cause the discontinuation of therapy, and the effects of five more years of treatment with this hormonal therapy, is required.

It is noteworthy that systematic reviews about neoadjuvant chemotherapy (NAC) are lacking. NAC represents a standard of care in many FBC cases but, for example, Leone et al. [44], comparing 385 MaBC and 68,065 FBC cases showed that pathologic complete response (pCR) in men was lower than for FBC patients. There are some possible explanations for the lower rate of pCR in MaBC with respect to FBC; for example, it may be due to the high prevalence of luminal disease in MaBC patients, which causes a lower sensitivity to NAC [58]. Another possibility could be represented by the different MaBC microenvironment in which there are lower rates of active T cells, predicting lower pCR rates to NAC [59]. Furthermore, it should be noted that this was not a prospective study and, in general, prospective trials about this topic are not available.

No evidence from systematic reviews is available for triple-negative breast cancer (TNBC). Although rare in men, this subtype is associated with a poorer prognosis due to its aggressive biological behavior and early metastatic potential. In female patients, NAC is recommended even for small tumors (cT1c cN0) or node-positive diseases (cN1 or more) as it enables tumor size reduction, increases the likelihood of breast-conserving surgery, and improves long-term survival outcomes. Recently, the addition of immunotherapy to NAC has marked a significant advancement in TNBC treatment. The KEYNOTE-522 trial demonstrated that the combination of pembrolizumab and chemotherapy significantly increases the pCR rate compared to chemotherapy alone, with a positive impact on OS [60].

For HER2-positive disease, NAC is the standard of care for tumors larger than 1.5–2 cm (cT1c cN0) or with nodal involvement (cN1 or more). The integration of chemotherapy with anti-HER2 agents, such as trastuzumab and pertuzumab, has been shown to increase the pCR rate and significantly improve prognosis by reducing the risk of recurrence. This approach has been validated by multiple studies, including the KRISTINE trial, which compared trastuzumab and pertuzumab combined with chemotherapy versus a strategy based solely on T-DM1 and pertuzumab, demonstrating the superiority of the chemotherapy-based combination in achieving pCR [61].

In the adjuvant setting, patients who do not achieve a pathological complete response after neoadjuvant therapy may benefit from trastuzumab emtansine (T-DM1), as demonstrated by the KATHERINE trial, which showed a significant reduction in the risk of recurrence or death compared to trastuzumab alone. Again, the evidence primarily comes from female populations, but it serves as the best available reference for guiding the treatment of HER2-positive MaBC [62].

Although specific data on (neo)adjuvant treatments in MaBC are limited, current clinical management follows the guidelines established for female patients. It is crucial that future studies include a larger number of male patients to determine potential differences in treatment response and optimize therapeutic strategies for this specific population.

Moreover, systematic reviews and prospective data for the therapy of metastatic MaBC are missing. In ASCO guidelines, endocrine therapy should be administrated as the first line in advanced or metastatic HR+ MaBC, while chemotherapy should be reserved in cases with visceral crisis or rapidly progressive disease [1].

An important gap that must be underlined is the lack of published systematic reviews on the use of cyclin-dependent kinase (CDK 4/6) inhibitors in systemic treatment in advanced-stage MaBC patients. Because of the rarity of MaBC, data on new therapeutic agents, such as CDK 4/6 inhibitors, are limited: phase 3 clinical trials have not been conducted, and treatment management is extrapolated from FBC patients. In large clinical trials conducted of metastatic HR+, HER2-metastatic FBC palbociclib, abemaciclib, and ribociclib led to the improvement of PFS [63,64,65,66,67]. In the literature, there are some case reports and retrospective studies conducted of MaBC patients [68,69]. For example, Yildirim et al. conducted [70] a multicenter retrospective study on 46 male patients with HR+ and HER2-breast cancer, treated with palbociclib or ribociclib, proving that these therapeutic agents are effective and safe options in these kinds of patients, and supporting the use of CDK 4/6 inhibitor-based combinations as standard treatment in males also.

In this umbrella review, there are some limitations. For example, the statistical power of some meta-analysis could be influenced and decreased by the small number of the included studies. Other potential biases are linked to differences in study design, patients, quality, methods, and the reference standards of the included manuscripts.

To minimize possible biases, two independent authors selected the articles, extracted data, and assessed the quality of the included studies.

## 5. Conclusions

The data of this umbrella review prove that:

− BCS is safe and represents a reasonable treatment approach in selected MaBC cases;
− SLNB represents a successful and accurate practice;
− Adjuvant radiotherapy, improving disease local control and OS, has to be considered following partial mastectomy and also after radical mastectomy, in cases of involved nodes;
− Tamoxifen improves survival outcomes and an aromatase inhibitor and gonadotrophin-releasing hormone (GnRH) should be used only in case of contraindications to tamoxifen.

The available data confirm some of the standard therapies, while some results or lacking data prove that additional research and specific treatment trials and systematic reviews of the published data and literature are needed to improve current guidelines for MaBC patients.

## Figures and Tables

**Table 1:** Summary of characteristics and findings of included articles.

First Author	Year	Studies Included	Total Patients Involved	With/Without Meta-Analysis	Main Findings
Jardel et al. [47]	2018	29	10,965	Without	RT is mandatory following partial mastectomy. After radical mastectomy it has to be considered in case of involved nodes. For other prognostic subgroups, especially in pN0, comparable women risk factors should be assessed, but evidence is not sufficient.
De La Cruz et al. [51]	2019	8	5864	Without	BCS represents a safe treatment in selected patients, without the impairment of LR or DFS in MaBC patients.
Sauder et al. [48]	2020	9	11,592	With	BCS, with similar oncologic outcomes with respect to those of mastectomy, represents a feasible and reasonable treatment approach.
Lin et al. [49]	2021	40	14,061 surgery 11.392 RT 213 SLNB 11.229 ET	With	Both MaBCS and mastectomy show comparable OS, DFS, and local control. Chest-wall post-mastectomy radiation increases the OS. SLNB is related to high identification and low false-negative rates. Tamoxifen treatment significantly increases OS.
Rutherford et al. [50]	2022	59	39,529	Without	Adjuvant therapies significantly improve survival outcomes. Genetic screening should be offered to all MaBC patients.
Parpex et al. [52]	2024	2	164	With	In MaBC cases of negative preoperative axillary assessment and primary surgery, SLNB is consistent, reproducible, and effective. The extemporaneous SLNB histologic evaluation is encouraged because it would make it possible to quickly perform ALND in the case of a confirmation of lymph node metastasis.
Colciago et al. [53]	2014	14	80,219	With	A notable clinical benefit on OS is observed when RT is included in the therapeutic algorithm of treatment of MaBC patients.

ALND: Axillary lymph node dissection; BCS: breast-conserving surgery; DFS: disease-free survival; ET: endocrine therapy; LR: local recurrence; MaBC: male breast cancer; OS: overall survival; RT: radiotherapy; SLNB: sentinel lymph node biopsy.

## Data Availability

No new data were created; all data have been collected from publicly available studies that can be consulted on the following site: https://pubmed.ncbi.nlm.nih.gov/.

## Supplementary Materials

The following supporting information can be downloaded at: https://www.mdpi.com/article/10.3390/jpm15020066/s1, Table S1: Quality assessment of the included systematic reviews. Reference [71] is cited in the Supplementary Materials.
