# Supplementary material for: Treatments of Interest in Male Breast Cancer: An Umbrella Review
Source: J Pers Med. 2025 Feb 11;15(2):66. doi: 10.3390/jpm15020066 (PMC11856642; doi:10.3390/jpm15020066)
Supplement: Supplementary file 1 [file jpm-15-00066-s001.zip › jpm-3445139-supplementary.pdf]

Quality assessment (AMSTAR 2)* of the included systematic reviews [71]

Items judged of critical importance: comprehensiveness of the literature search; included studies and reasons for excluding studies described in adequate detail; adequacy of risk of bias assessment of primary studies; appropriateness of meta-analytical methods (only when a meta-analysis is performed); consideration of risk of bias when interpreting the results of the review. According to the responses to critical items, a final overall judgment of the whole review can be:

High quality: No or one non-critical weakness: the systematic review provides an accurate and comprehensive summary of the results of the available studies that address the question of interest

Moderate quality: More than one non-critical weakness but no critical flaws. It may provide an accurate summary of the results of the available studies that were included in the review

Low quality: One critical flaw with or without non-critical weaknesses: the review may not provide an accurate and comprehensive summary of the available studies that address the question of interest

Critically low quality: More than one critical flaw with or without non-critical weaknesses: the review should not be relied on to provide an accurate and comprehensive summary of the available studies

* [71] Shea, B.J.; Reeves, B.C.; Wells, G.; Thuku, M.; Hamel, C.; Moran, J.; Moher, D.; Tugwell, P.; Welch, V.; Kristjansson, E.; et al. AMSTAR 2: A critical appraisal tool for systematic reviews that include randomised or non-randomised studies of healthcare interventions, or both. *BMJ* **2017**, *358*, j4008.

Table S1. Quality assessment of the included systematic reviews

n	AMSTAR-2 criteria	**Jardel et al.**	**De La Cruz et al.**	**Sauder et al.**	**Lin et al.**	**Rutherford et al.**	**Parpex et al.**	**Colciago et al.**
1	Research questions and inclusion criteria include components of PICO	yes	yes	yes	yes	yes	yes	yes
2	Review methods established prior to the conduct of the review (protocol) and deviations justified	yes	yes	yes	yes	yes	yes	yes
3	Selection of study design explained	yes	yes	yes	yes	yes	yes	yes
4	Comprehensive literature search strategy	no (PubMed only)	no (PubMed only)	yes	yes	yes	yes	yes
5	Study selection in duplicate	not reported	yes	yes	yes	yes	yes	yes
6	Data extraction in duplicate	not reported	yes	yes	yes	yes	yes	yes
7	List of excluded studies and justification of the exclusions	yes	yes	yes	yes	yes	yes	yes
8	Included studies described in adequate detail	yes	yes	yes	yes	yes	yes	yes
9	Technique for assessing the risk of bias satisfactory	yes	partial yes	yes	yes	no	yes	yes
10	Sources of funding for the primary studies reported	yes	yes	yes	yes	not reported	yes	yes
11	Appropriate methods for meta-analysis	no meta-analysis conducted	no meta-analysis conducted	yes	yes	no meta-analysis conducted	yes	yes
12	Potential impact of risk of bias results on meta-analysis assessed	no meta-analysis conducted	no meta-analysis conducted	yes	yes	no meta-analysis conducted	yes	yes
13	Risk of bias results accounted for in discussion/conclusion	yes	yes	partial yes	yes	yes	yes	yes
14	Satisfactory discussion and explanation of observed heterogeneity, if any	yes	yew	partial yes	yes	partial yes	yes	yes
15	Adequate investigation of publication bias	no meta-analysis conducted	no meta-analysis conducted	yes	yes	no meta-analysis conducted	yes	yes
16	Conflict of interest of review authors and funding received for conducting the review reported	yes	yes	yes	yes	not reported	yes	yes
	Overall methodological quality	**low**	**low**	**moderate**	**high**	**low**	**high**	**high**
